# Immigration, acculturation, and preferred help-seeking sources for depression: comparison of five ethnic groups

**DOI:** 10.1186/s12913-020-05478-x

**Published:** 2020-07-11

**Authors:** Valeria Markova, Gro M. Sandal, Ståle Pallesen

**Affiliations:** 1 Department of Pulmonology, Haukeland University Hospital, Bergen, Norway; 2 Department of Psychosocial Science, University of Bergen, Bergen, Norway; 3 Optentia Research Focus Area, North-West University, Vanderbijlpark Campus, Vanderbijlpark, South Africa

**Keywords:** Depression, Ethnic groups, Minority groups, Acculturation, Help-seeking behavior, Immigrant, Refugees, Mental health services, Help-seeking intentions, Vignette methodology

## Abstract

**Background:**

Immigrants are more likely than the majority population to have unmet needs for public mental health services. This study aims to understand potential ethnic differences in preferred help-seeking sources for depression in Norway, and how such preferences relate to acculturation orientation.

**Methods:**

A convenience sample of immigrants from Russia (*n* = 164), Poland (*n* = 127), Pakistan (*n* = 128), and Somalia (*n* = 114), and Norwegian students (*n* = 250) completed a survey. The sample was recruited from social media platforms, emails, and direct contact. The survey consisted of a vignette describing a moderately depressed person. Respondents were asked to provide advice to the person by completing a modified version of the General Help-Seeking Questionnaire. The immigrant sample also responded to questions about acculturation orientation using the Vancouver Index of Acculturation Scale.

**Results:**

Significant differences were found in the endorsement of traditional (e.g., religious leader), informal (e.g., family), and semiformal (e.g., internet forum) help-sources between immigrant groups, and between immigrant groups and the Norwegian respondent group. Immigrants from Pakistan and Somalia endorsed traditional help sources to a greater extent than immigrants from Russia and Poland, and the Norwegian student sample. There were no ethnic differences in endorsement of formal mental help sources (e.g., a medical doctor). Maintenance of the culture of origin as the acculturation orientation was associated with preferences for traditional and informal help sources, while the adoption of mainstream culture was associated with semiformal and formal help-seeking sources.

**Conclusion:**

Ethnic differences in help-seeking sources need to be considered when designing and implementing mental health services.

## Background

Providing efficient mental health services for a growing immigrant population is a significant challenge for many countries. Acculturative stress, low socio-economic status, social isolation, and feelings of powerlessness in the country of settlement are factors that are recognized to increase the vulnerability of immigrants to mental health problems [1–4]. For refugees, trauma experienced before and during their flight may also have severe consequences for their mental health [1, 5, 6]. A nationwide cross-sectional study in Norway reported that immigrants had a higher likelihood of being frequent attenders at general practitioners (GP) than the native population. Problems related to mental health were one of the most common factors associated with frequent visits among immigrants from low- and middle-income countries [7]. While increased attention to access to mental healthcare has been seen among immigrants in recent years [5, 8, 9], epidemiological research from Norway and other European countries suggest underutilization of specialized mental health services among some immigrant groups compared to the native population [1, 2, 8–11]. The rate has been found to vary widely by country of origin [12, 13]. This might imply that some immigrant groups have a higher proportion of untreated mental health problems than the rest of the population or that help is sought from sources outside the public health system. Understanding the help-seeking pattern for mental health problems among immigrant populations is important if research, policy, and tailored health program initiatives are to reach vulnerable or isolated groups. Our focus in this paper is on help-seeking for depression because of its high prevalence, and comorbidity with other common diseases [14].

Although there are many definitions of help-seeking [15], it is defined here as a request for assistance from formalized services or for informal support for the purpose of resolving emotional, behavioral, or health problems [16]. From a public health perspective, the proposed Behavioral Model of Health Service Use [17] is useful for understanding ethnic differences in help-seeking preferences [17, 18]. According to this model [17, 18], three interrelated groups of factors influence all health behavior, including help-seeking behavior: predisposing factors (e.g., gender, ethnicity, and socio-economic status); need factors (e.g., self-perceived need and professional evaluation), and enabling factors (e.g., ability to pay for healthcare, health literacy, and social support). The model suggests that health behaviors are continuously re-defined by experience and that they influence all health outcomes. Based on a systematic review, Malgaard and colleagues [19] concluded that belonging to certain ethnic minority groups represented a risk of not seeking professional help for depression (based on U.S. and Canadian data sets). For example, African Americans and Mexican Americans had lower rates of seeking help for major depression compared to those with a Caucasian background. Differences were attributed to mental health literacy and attitude-related barriers such as shame [5]. With a view to improving ethnic minority patients’ access to care, three recent review papers on mental help-seeking behavior [5, 19, 20] highlight that further research should explore beliefs about what constitute appropriate sources of care and help-seeking for mental health concerns in specific ethnic and religious minorities groups.

It is a widely held assumption that the more immigrants integrate into the dominant culture of their country of settlement, the more they will adopt the health patterns of the majority [21]. In line with this, we expect acculturation to be an important variable in terms of understanding individual variations in help-seeking within immigrant groups. Acculturation is defined as the changes in values and behaviors individuals make to accommodate to the culture of settlement [22]. Berry [22] argued that acculturation addresses two underlying dimensions: the degree to which one’s heritage culture is maintained and the degree to which one wishes to participate and have contact with other cultural groups. This two-dimensional perspective implies that immigrants can maintain or neglect their home culture, while simultaneously adopting or not adopting the culture of settlement [22]. Thus, immigrants may retain traditional help-seeking patterns from their home culture despite long residence time and adoption of the majority culture in other domains. In line with previous research [21, 23], there is reason to assume that immigrants who adopt the majority culture are likely to be more positive about seeking help from public health services (formal sources) than immigrants who do not. However, studies on acculturation orientation and help-seeking are few and divergent, and they have mainly concerned Asian-American immigrant groups in the US [23]. More research has been called for on specific migrant groups and how they view mental illness [24]. Because immigrant groups differ significantly between and within themselves as regards enabling, predisposing, and need factors, differences within immigrant groups are as interesting as differences between immigrant groups and the native Norwegian population. Previous research has shown that several factors can influence acculturation orientation, most importantly gender, and length of time abroad [23].

Against this backdrop, this study aims to examine and compare preferred help-seeking sources for depression among different immigrant groups (Poles, Russians, Somalis, and Pakistanis) in Norway and how such preferences relate to acculturation orientation. The immigrant groups were chosen because they are among the largest immigrant groups in Norway [8]. At the group level, they also differ in terms of years lived in Norway, the reason for migration, and religious orientation. In this paper, the term “immigrants” is defined as persons who have either immigrated to Norway themselves or were born of two non-Norwegian-born parents. We focus on lay people instead of a clinical population. Lay people refer to persons who are not mental healthcare professionals. The high prevalence of depression suggests that many people will experience this disease either themselves or their family members will. Research suggests that, particularly in communal cultures, the views of family members will strongly influence the choice of help-seeking sources [25]. Thus, the understandings of lay people may be highly informative about how immigrants experience and cope with depression.

Recruiting a representative sample of migrants to surveys is challenging. In the present study digital social platforms (Facebook, LinkedIn), emails, and direct contact were used to target and recruit participants. This approach was combined with snowball sampling, that is, immigrants interested in participating in the study were asked to encourage the participation of immigrants from the same country of origin. Although this flexible approach resulted in a convenience sample, it was preferred to more traditional techniques such as population register-based sampling and onomastic (name-based) sampling [26–28]. Registers are often incomplete when it comes to foreign citizenship, and linguistic screening of names have obvious shortcomings as they frequently cannot identify ethnic group membership with certainty. Vulnerable immigrant groups may be reluctant to participate in surveys for several reasons ranging from the experience of persecution and mistrust of authorities in their countries of origin to cultural issues and language skills. The choice of recruitment method in the present study was based on an assumption that immigrants would be more willing to participate if members of their ethnic group encouraged participation.

## Methods

### Sample and study participants

A convenience sample of 533 respondents was recruited from four immigrant groups in Norway. In addition, data from Norwegian students (*N* = 250) were used as a native comparison in parts of the analyses. In total, 81 respondents had more than 30 missing data points (out of 783 responses; 10%) and were excluded from all statistical analyses. Hence, the final sample consisted of 702 participants. The age of the respondents ranged from 19 to 64 years with a mean of 30.8 (*SD* = 9.3). Power analysis was conducted with G*Power, version 3.0.3 [29]. Setting alpha to .05 (two-tailed), power (1-β) to .80, and effect sizes (Cohens *d*) to 0.2 (small), 0.5 (medium), and 0.8 (large) comparing five groups shows that a total of 1200, 200 and 80 respondents were needed, respectively. As we recruited about 100 subjects from each group, we were accordingly able to detect medium and larger effect sizes.

### Procedure

#### Immigrant samples

The survey was distributed and collected on paper (*n* = 33) or online (*n* = 500). The possibility of answering the survey on paper was only offered to the Somali respondents. Some of the data on Somali immigrants have been presented in a previous paper [30]. As for the online survey, the respondents were recruited through social network sites (e.g., Facebook, online immigrant organizations). Only those who actively expressed consent received the online link or the paper version of the survey. Prior to answering the questionnaires, respondents were informed that the study aimed to provide more knowledge about how people from different cultures think that one should best deal with feelings such as sadness and that such knowledge could inform the development of health services adapted to the needs of minority groups. Respondents were also informed about how data would be handled in all phases of data collection and publication. Data were collected by the first author and four researcher-assistants with origin in Russia, Poland, Somalia, and Pakistan. Respondents with Somali and Pakistani origin could choose to answer the survey in either Arabic, English, or Norwegian. Respondents with Russian and Polish origin could in addition, choose to answer the survey in Russian and Polish, respectively. The instruments were translated using a translation-back-translation procedure, comparing versions to maximize technical, semantic, content, and conceptual equivalence.

#### The Norwegian sample

The survey was distributed online. A research assistant invited respondents via a private message on Facebook or by email. The students were mainly recruited from higher education institutions in Norway. Except for psychology students who were not recruited due to their professional training background in mental health care, students were invited to participate in the study independent of their academic discipline. The student sample represented different academic disciplines: 30% humanities (e.g., pedagogy), 30% social sciences (e.g., psychology), 11% natural sciences (e.g., chemistry), 16% medicine (e.g., nursing) and 13% from formal science disciplines and professions (e.g., law and real estate management). Students (*n* = 25) who had migrated from other countries were not included in the analysis.

### Instruments

The first part of the survey consisted of questions about demographics, including age, gender, ethnicity, years of formal education, and length of residence in Norway. Respondents were then asked to read a vignette (Table 1), describing a person with symptoms of depression consistent with the criteria for a depressive episode in the International Classification of Diseases-10 [31]. The gender of the vignette character was matched to the respondent to facilitate identification.

**Table 1:** Vignette used in the survey

“*John/Ann is a 27-year-old waiter in a restaurant in Bergen. He/she was born in Oslo to parents who were restaurant owners, but has made Bergen his/her home for 5 years. In the last few weeks, he/she has been experiencing feelings of sadness every day. John/Ann’s sadness has been continuous, and he/she cannot attribute it to any specific event or to the season of the year. It is hard for him/her to go to work every day; he/she used to enjoy the company of his/her co-workers and working at the restaurant, but now he/she cannot find any pleasure in this. In fact, John/Ann has little interest in most activities that he/she once enjoyed. He/she is not married and lives alone, near his/her brother/sister. Usually, they enjoy going out together and with friends. But now he/she does not enjoy this anymore. John/Ann feels very guilty about feeling so sad, and feels that he/she has let down his/her brother/sister and friends. He/she has tried to change his/her work habits and start new hobbies to become motivated again, but he/she cannot concentrate on these tasks. Even his/her brother/sister has now commented that John/Ann gets distracted too easily and cannot make decisions. Since these problems began, John/Ann has been sleeping poorly every night; he/she has trouble falling asleep and often wakes up during the night. A few nights ago, as he/she lay awake, trying to fall asleep, John/Ann began to cry because he/she felt so helpless*.”	

In the Russian version, the male name John was changed to the more typical Russian name Zenia

After reading the vignette, the respondents answered questionnaires about help-seeking preferences and acculturation orientation.

*The General Help-Seeking Questionnaire* [30, 32] (GHSQ) consists of 19 items describing different sources from whom help can be sought (e.g., friends, traditional healer, and telephone helpline). Each item was rated on a six-point Likert scale (1 = “very unlikely” to 6 = “very likely”). The standard instruction: “*If you were having [problem-type]*, *how likely is it that you would seek help from the following people?*” [32], was modified to: “*If you were feeling like Ann/John (gender-matched)*, *how likely is it that you would seek help from the following sources?*”. In line with the recommendations of Wilson et al. [32], relevant items were added to fit the target group. Specifically, we included items referring to help-seeking sources in the immigrant community (e.g., traditional healers, elders in my community, leaders in my ethnic community or from the same country as me, other people in my ethnic community or from the same country as me) and alternative medicine (e.g., acupuncture, homeopathy). One source (the Norwegian Labor and Welfare Administration, abbreviated to Social Worker/NAV in the survey) was added to adapt the questionnaire to the Norwegian context.

*The Vancouver Index of Acculturation* [33] *(*VIA) measures acculturation orientation. It consists of 20 statements assessing interest and participation in one’s heritage culture (10 items) and the mainstream (Norwegian) culture in the country of residence (10 items). Each item was rated on a nine-point Likert scale (1 = “strongly disagree” to 9 = “strongly agree”). The average of the 10 items in each subscale was computed, resulting in a score for each participant on the heritage subscale and on the mainstream subscale. These scales are in the following referred to as “*Maintenance*” and “*Adoption*”.

### Data analysis

SPSS 24.0 was used for all statistical analyses. A parallel principal component analysis (with Varimax rotation) of all items in the GHSQ was conducted of help-seeking sources that tend to be used simultaneously. Items with cross-loadings of .40 or higher on two or more factors were removed [34]. Based on the results, composite scores for the subscales were computed for each factor. Secondly, differences in means between all immigrant groups were assessed using a multivariate analysis of variance (MANOVA) and Tukey post-hoc tests. Thirdly, a correlation analysis was conducted to explore the relationship between preferred help-seeking sources, acculturation orientation (only immigrants), and background variables. Finally, a hierarchical multiple regression analysis was conducted to investigate whether the acculturation subscales explained help-seeking preferences when controlling for gender, age, years of higher education and ethnicity in the immigrant sample. Age was controlled for by partial correlation analysis and an analysis of covariance (ANCOVA), and no significant differences were observed (results not shown).

## Results

### Descriptive statistics

Table 2 shows the demographic characteristics of the different subsamples.

**Table 2:** Descriptive statistics for the samples

Country of origin	Norway (*n* = 225)	Russia (*n* = 151)	Poland (*n* = 109)	Pakistan (*n* = 117)	Somalia (*n* = 100)
	*M (SD)*	*M (SD)*	*M (SD)*	*M (SD)*	*M (SD)*
Age	27.3 (7.0)	34,8 (8.5)	34.4 (9.6)	28.5 (10.2)	28.9 (8.3)
Years in Norway	Not relevant	7.9 (5.2)	6.1 (5.8)	16.7 (8.8)	9.3 (7.1)
Born in Norway (*N*)	Not relevant	11	2	86	0
Higher education^a^	100%	79%	79%	80%	35%
Females	69%	87%	77%	69%	44%

^a^Includes those who have started, are undertaking or have completed studies at the university level

#### Factor structure of the GHSQ

A principal component analysis (Table 3) yielded four factors with eigenvalues exceeding 1, accounting for 57% of the total variance. A scree plot and parallel analysis both supported the 4-factor solution. Two items were deleted due to cross-loadings (“*I would not seek help from anyone”* and “*I would seek help from my manager or human resource staff at my workplace”*), and one item (“*I would seek help from social worker/NAV*”) was deleted because the content diverged from the other items with high loading on the factor. Fifteen items were included in further analyses. Bartlett’s test of sphericity was significant, and the Kaiser-Mayer-Olkin measure of sampling was acceptable (≤ .81). The first factor, explaining 26% of the variance, covered help-seeking from religious leaders, healers, elders, and members of the ethnic community. This factor was labelled *traditional*. The second factor, explaining 13% of the variance, included family members, friends, and partners. This factor was labelled *informal*. The third factor, explaining 10% of the variance, concerned phone helplines, internet forums, and a work colleague, and was labelled *semiformal*. The fourth factor, explaining 8% of the variance, comprised general practitioners and psychiatrists/psychologists and was labelled *formal*. The same analysis of only the immigrant sample resulted in a similar factor structure.

**Table 3:** Factor loadings for parallel principal component analysis with varimax rotation of help-seeking questionnaire

	Traditional	Informal	Semiformal	Formal
Leader in my ethnic community or from the same country as me	**.84**	.09	.20	−.04
Elders in my community	**.80**	.15	.12	−.01
Traditional healer	**.76**	−.00	.14	.08
Other people in my ethnic community or from the same country as me	**.75**	.13	.18	−.08
Religious leader (e.g., priest, rabbi, chaplain, mullah)	**.72**	.15	.04	−.02
Alternative medicine (e.g., acupuncturist, homeopath)	**.60**	−.04	.08	.26
Parents	.18	**.75**	−.04	.01
Friends	.04	**.74**	.14	.05
Intimate Partner (e.g., girlfriend, boyfriend, husband, wife)	−.13	**.72**	−.01	.15
Other relative/Family member	32	**.61**	.09	.05
Telephone helplines	.24	−.03	**.75**	.20
Internet forums	.03	−.03	**.79**	.09
Work colleague	.28	.36	**.56**	−.02
Psychiatrist/psychologist	−.01	.04	.10	**.85**
Medical doctor/GP	.11	.19	.13	**.78**

Items loaded under the same factor in boldface

#### Differences across ethnic groups in health-seeking sources

The results from the MANOVA with Tukey’s post-hoc tests, with factor scores as dependent variables and ethnic group affiliation as an independent variable, are presented in Table 4. Preliminary assumption testing was conducted to check for normality, linearity, univariate and multivariate outliers, and multicollinearity, with no serious violations being noted. Levene’s test showed, however, that the assumption of equality was violated. In line with the recommendations of Tabachnick and Fidell [35], a more conservative alpha (.025) level was therefore used. Three of the help-seeking factors varied significantly between ethnic groups: traditional help-seeking (*F*_4,697_ = 65.18, *p <* 0.001), informal help-seeking (*F*_4,697_ = 7.66, *p* < 0.001), and formal help-seeking (*F*_4,697_ = 3.20, *p* < 0.025). Specifically, the Somali respondents showed a stronger preference for traditional help-seeking than respondents from the other ethnic groups. Post-hoc tests indicated that the mean score for the Traditional factor for respondents of Somali origin was significantly different from the Pakistani immigrant sample, with a moderate effect size (*d* = 0.64), and the Russian immigrant sample (*d* = 0.99), Polish immigrant sample (*d* = 1.24), and Norwegian student sample (*d* = 1.32), with large effect sizes. The mean score of the Pakistani immigrant sample on the Traditional factor was significantly different from the Russian immigrant sample (*d* = 1.22) and Norwegian student sample (*d* = 0.87), with large effect size, and from the Polish immigrant sample (*d* = 0.69), with a moderate effect size. The Russian immigrant sample was significantly different from the Norwegian student sample, with a moderate effect size (*d* = 0.57). All immigrant samples and the Norwegian students scored highest on the Informal factor relative to the three other factors. Respondents of Somali origin scored higher on the Informal help-seeking factor than the Pakistani (*d* = 0.40), Russian (*d* = 0.43), and Polish immigrant samples (*d* = 0.62) and the Norwegian student sample (*d* = 0.61), with moderate effect sizes. Scores on Formal help-seeking also varied significantly between ethnic groups, but post-hoc tests show no significant results.

**Table 4:** MANOVA – Differences in help-seeking strategies based on ethnic groups (factor level)

Country of origin	Norway	Russia	Poland	Pakistan	Somalia	*F* (*4,729*)	*p*	Partial Eta Square
	*M* (*SD*)	*M* (*SD*)	*M* (*SD*)	*M* (*SD*)	*M* (*SD*)			
Traditional	1.39 (0.64)^a^	**1.78 (0.73)**^b, c^	**1.51 (0.73)**^a, b^	**2.09 (0.94)**^c^	**2.57 (1.26)**^d^	**65.17**	**< 0.00**	**0.24**
Informal	3.80 (1.12)^a^	4.03 (0.97)^a^	3.78 (1.14)^a^	4.04 (1.17)^a^	**4.50 (1.13)**^**b**^	**7.65**	**< 0.00**	**0.04**
Semiformal	2.24 (0.87)^a, b^	2.31 (0.95)^a, b^	2.15 (1.07)^a^	2.17 (0.98)^a, b^	2.34 (1.14)^b^	2.55	0.04	0.01
Formal	3.62 (1.33)^a^	3.31 (1.36)^a^	3.37 (1.51)^a^	3.22 (1.30)^a^	3.35 (1.44)^a^	3.02	0.01	0.02

Means within a row with different subscripts are significantly different at *p* ≤ .025

#### Help-seeking factors in relation to acculturation orientation and demographic variables

The correlational analysis (Table 5) showed that endorsement of traditional and informal help-seeking sources was positively associated with a maintenance acculturation orientation, while endorsements of semiformal and formal help-seeking sources were positively associated with an adoption acculturation orientation. This indicates that acculturation orientation may influence help-seeking preferences. Higher education correlated negatively with the endorsement of traditional help-seeking sources and positively with adoption acculturation orientation and age. There were also significant gender differences. Men endorsed traditional help-seeking sources more than women, whereas women endorsed formal help-seeking sources more often than men.

**Table 5:** Mean, standard deviations, and correlations between help-seeking strategies, acculturation orientation and demographic characteristics

	*M*	*SD*	*1*	*2*	*3*	*4*	*5*	*6*	*7*	*8*
*Help-seeking*										
1.Traditional	1.80	0.96	**.84**							
2. Informal	4.00	1.10	.26**	**.71**						
3.Semiformal	2.30	1.01	.31^**^	.20^**^	**.60**					
4. Formal	3.46	1.40	.08^*^	.14^**^	.22^**^	**.61**				
*Acculturation*^a^										
5. Maintenance	6.57	1.70	.27^**^	.31^**^	.06	.05	**.90**			
6. Adoption	5.60	1.70	−.06	.05	.16^**^	.14^**^	.19^**^	**.90**		
*Demographic*										
7. Gender^b^	30.80	9.30	−.09^*^	.03	−.01	.14^**^	.02	.05		
8. Age	2.77	1.30	.06	−.10^**^	−.00	.02	−.03	−.07	.05	
9. Higher education	1.70	0.46	−.18^**^	−.00	.01	−.04	−.05	.22^**^	.07	.24^**^

*Note.* The coefficients on the diagonal in bold are the Cronbach’s alpha of each scale

^a^Only immigrant sample (*n* = 452). ^b^1 = male, 2 = female

* *p* ≤ .05, ** *p* ≤ 0.01 (2-tailed)

Finally, a hierarchical multiple regression analysis was carried out (see Table 6). Since different approaches to help-seeking are associated with age, gender, education level, and ethnicity [17, 19, 23] we controlled for these factors in the regression analysis when investigating the impact of acculturation orientation on types of help-seeking behavior. Demographic variables were entered in the first block, followed by the acculturation orientation factors of maintenance and adoption, which were entered in the second block. Russian immigrant sample was used as the contrast/reference group in the regression analyses. Missing data were dealt with by pairwise deletion. Gender accounted for a significant variance. Female respondents endorsed formal help-seeking sources more than men. The results also show that the Somali respondents preferred traditional and informal help-seeking sources more than the contrast/reference group. The Polish respondents preferred traditional help-seeking sources to a smaller degree than the contrast/reference group.

**Table 6:** Summary of results from hierarchical multiple regression analyses (*N* = 452)

	**Traditional**				**Informal**			
	*ƅ*	β	95% Cl	*p*	*ƅ*	β	95% Cl	*p*
Step 1								
Gender^a^	-.12	-.06	-.32, .08	.225	.18	.07	-.06, .43	.147
Age	.10	.10	.00, .02	.029	-.01	-.06	-.02, .00	.212
Education^b^	-.15	-.19	-.22, -.07	**<.000**	.01	-.08	-.11, .09	.890
Somalia	.69	.28	.40, .99	**<.000**	.44	.15	.06, .81	.**022**
Pakistan	.22	.10	-.02, .47	.076	.00	.00	-.31, .31	.989
Poland	-.37	-.16	-.61, -.13	**.002**	-.27	-.10	-.57, .04	.095
R²		.22		<.**000**		05		**.001**
Step 2								
Gender	-.15	-.07	-.34, .05	.138	.15	.06	-.09, .39	.215
Age	.01	.01	.00, .02	.034	-.01	-.07	-.02, .00	.183
Education	-.14	-.18	**-**.22, -.06	**<.000**	-.01	-.01	-.10, .09	.901
Somalia	.61	.25	.32, .91	<.**000**	.34	.12	-.03, .70	.070
Pakistan	.21	.09	-.03, .45	.083	-.01	-.00	-.31, .29	.940
Poland	-.34	-.14	-.57, -.10	.**005**	-.19	-.07	-.49, .10	.201
Maintenance	.13	.03	.08, .18	<.**000**	.19	.28	.13, .26	<.**000**
Adoption	-.02	.03	-.07, .03	.478	-.00	-.00	-.07, .06	.986
∆R^2^		.04		<.**000**		.08		**<.000**
Total R²		.28		<.**000**		.13		**<.000**
	**Semiformal**				**Formal**			
	*ƅ*	β	95% Cl	*p*	*ƅ*	β	95% Cl	*p*
Step 1								
Gender^a^	.03	.01	-.20, .28	.788	.61	.20	.31, .91	**<.000**
Age	-.00	-.03	-.02, .01	.524	.01	.08	-.00, .03	.128
Education^b^	-.01	-.02	-.11, .08	.787	.01	.01	-.11, .12	.917
Somalia	-.01	-.01	-.38, .35	.943	.38	.11	-.07, .83	.099
Pakistan	-.14	-.06	-.44, .16	.363	.20	.06	-.17, .58	.283
Poland	-.15	-.06	-.44, .15	.331	.30	.09	-.07, .66	.110
R²		.01		.889		.04		.003
Step 2								
Gender	.04	.02	-.20, .28	.737	.62	.20	.33, .92	<.**000**
Age	-.00	-.02	-.01, .01	.768	.01	.10	.00, .03	.054
Education	-.04	-.05	-.13, .06	.437	-.03	-.03	-.14, .01	.647
Somalia	.07	.02	-.30, .43	.727	.49	.14	.04, .93	**.**056
Pakistan	-.13	-.05	-.43, .17	.387	.22	.07	-.15, .58	.247
Poland	-.05	-.02	-.35, .24	.733	.42	.13	.06, ..78	.063
Maintenance	.01	.02	-.05, .08	.675	.01	.01	.-.07, .09	.767
Adoption	.12	.18	.05, .18	<.**000**	.16	.19	.08, .24	<.**000**
∆R^2^		.03		.**001**		.063		<.**000**
Total R²		.04		.**001**		.08		<.**000**

^a^1 = male, 2 = female; ^b^1 = no higher education, 5 = Ph.D. level

## Discussion

The overall aim of this study was to examine and compare preferred help-seeking sources for depression among different immigrant groups (Poles, Russians, Somalis, and Pakistanis) in Norway, and to provide more insight into how such preferences relate to individual differences in acculturation orientation. Factor analysis suggested four main categories of help-seeking sources, labelled *traditional, informal, semiformal, and formal.* A similar classification into informal, semiformal, and formal help-seeking sources was suggested by Rickwood and Thomas [15] following a systematic review. They noted that classifications are not absolute, since different countries have different health and social care systems. For example, traditional healers could be a critical source of formal health care in a traditional indigenous population group. In the present study, traditional sources emerged as one distinct factor, comprising help-seeking from religious leaders, alternative medicine providers, and ethnic community members. It is also possible that the emergence of the semiformal help-seeking factor (that included internet forums), was influenced by the fact that the majority of participants being recruited from social media and therefore likely to be familiar with using digital platforms.

The results indicate that independent of ethnicity, respondents preferred to rely on informal sources of help, such as friends and family, before turning to semi-formal (e.g., telephone helplines) or formal (psychologists/psychiatrists and general practitioners) help sources. This is in line with previous research [36, 37] highlighting the importance of social networks in coping with mental health problems. Surprisingly, and contrary to previous studies [11, 38, 39], there were no differences between ethnic groups in preferences for formal help-seeking sources. This is an important finding since earlier research has indicated that some ethnic groups may have a lower preference for formal sources of help due to lower mental health literacy [40]. Our findings indicate that all groups recognize formal sources of help as valuable. One possible explanation for these different findings is that all legal residents in Norway have access to public health care and that costs are low. All citizens are entitled to a general practitioner. Once a person reaches an annual limit (currently about NOK 2000), services are free. However, when interpreting the findings, it should be kept in mind that some immigrants, in particular from countries where mental health services are sparse or non-existent, may not have a clear understanding of what a psychologist is or the nature of psychological treatment. Moreover, one should be mindful that the formal help factor only consisted of two items, which may explain why the internal consistency was rather low. We cannot rule out that the introduction provided to the respondents when they were invited to participate could have made them more inclined to endorsing formal help sources as they were informed that the study concerned how “one could best deal with feelings such as sadness” and that “the results could inform the development of health services adapted to the needs of minority groups”. According to Wright, Jorm, and Mackinnon [41] labelling a disorder has implications for help-seeking preferences and beliefs. However, the term depression was deliberately not used in the information provided to the participants. Therefore, we overall regard it unlikely that the instructions impacted substantially on the results.

Immigrants and refugees from Somalia and Pakistan endorsed more traditional and informal sources of help than immigrants from countries culturally closer to Norway (Russia and Poland) and the Norwegian sample. Thus, as the cultural distance grows, the conceptualization of what constitutes effective help-seeking sources seems to diverge. If informal and traditional sources are influential in determining treatment choices in depressed friends and family, this may highlight their potential role as gatekeepers or gate-openers for public mental health services [36]. The Norwegian student sample scored significantly lower than most ethnic groups on preference for traditional sources of help. This is consistent with previous research [42]. However, the lower endorsement of help-seeking from traditional sources may be due to the possible perceived irrelevance to the Norwegian respondents of some of the questions loading on the traditional factor (e.g., “*seeking help from a leader in my ethnic community or from the same country as me*”).

The results of the hierarchical regression analysis showed that acculturation orientation explained only a modest portion of the variance in preferred help-seeking sources. However, the pattern of correlations was in accordance with previous findings [38, 43]. Orientation towards heritage culture was associated with a preference for traditional and informal sources of help, while orientation towards mainstream (Norwegian) culture was associated with endorsement of semiformal and formal sources of help.

The current findings suggest that demographic variables should also be taken into consideration when designing interventions for immigrants. Women took a more positive view of formal help-seeking sources, while males took a more positive view of traditional help-seeking sources [19]. There may be several explanations for these findings, for example, the stigma attached to mental health among male respondents that have been reported in previous findings [20, 44]. Years of higher education was positively associated with endorsement of formal sources and negatively associated with endorsement of traditional help-seeking sources. These findings suggest that immigrants with lower education are more likely to seek help from sources outside the existing health services. This may give cause for concern because lower education, often associated with lower socio-economic status, is a risk factor for poorer mental health.

### Methodological considerations

Our results should be interpreted in light of certain limitations. The use of a vignette is useful in studies of nonclinical populations to attempt to determine what people who are not experiencing symptoms would do if they were to experience symptoms [15]. This approach may also have reduced the impact of social desirability since the respondents were not asked to report their mental health behavior. It can still be questioned whether the response to the question of what a hypothetical person should do reflects how the respondents themselves would have acted if they or someone in their family were depressed. Issues related to the representativeness of the samples need to be kept in mind. Participants in the present study were recruited through convenience sampling, primarily via emails, social media, and through snowball sampling. The latter sampling method is recommended when working with hard-to-reach population groups, such as ethnic minorities [26, 27]. In terms of recruitment through social media, it has been noted that this may lead to a mismatch between the target population and those recruited, especially regarding demographic variables [45]. The use of social media for recruitment may thus explain the preponderance of young participants. Taken together, lack of familiarity with social media platforms, low reading literacy, lack of acquaintance with questionnaires, and access to the internet are factors likely to have prevented participation in the study.

Readers should be mindful that these and other factors may influence the comparability of the samples. Ethnic differences in response styles [46] represent a possible bias, but we believe that the influence of this possible bias was minor as there was no evidence suggesting that specific ethnic groups consistently scored higher or lower on the scales. Educational attainment varied much between the groups. For the Pakistani, Polish and Somali samples, the portion with higher education are close to being representative for these immigrant groups in Norway, but in the Russian sample, a larger portion had higher education compared to the Russian immigrant population in Norway [47]. Also, the Norwegian students were on average somewhat younger and had a higher education level than the other groups. Except for the Somali immigrants, females were in the majority. To control for the possibility that gender, age, or level of education or interactions between these variables are responsible for the differences between the ethnic groups observed, we adjusted for these variables in the regression analyses. We acknowledge that it may be problematic to generalize from students to the general public [48]. Nonetheless, in Norway, the population’s education level is high, particularly in the younger age groups. Among those aged 15–64 years in 2019, 38% have a university or college degree compared to 28% in the European Union [49]. As a general rule, higher education is free of charge and Norwegian students are entitled to loans and grants from the State Educational Loan Fund. Therefore, the student population in Norway is probably more heterogeneous regarding backgrounds than in many other countries [50]. Recent research on Norwegian students enrolled in higher education showed higher levels of mental health problems than the general population [51]. The high prevalence of mental health problems in university students correspond with data from other countries [52], indicating that this group may be particularly relevant when examining help-seeking behavior for mental health problems.

## Conclusion

Future studies are needed to understand the mechanisms underlying ethnic differences in help source preferences, as well as to enable the generalization of the results from this study to more heterogeneous populations. Nonetheless, the results from this study suggest that immigrants’ preferred help-seeking sources differ by ethnic group, gender, level of education, and acculturation orientation. The differences were particularly evident as regards choosing traditional help-seeking sources. One implication of the findings is that public health services for ethnic minority patients, in particular for men and those with lower education, should consider integrating formal, informal, and traditional help sources, such as ethnic community members, religious leaders, and family networks when designing and implementing mental health services.

## Data Availability

The datasets used for the current study are available from the corresponding author upon request.

## Abbreviations

VIA: The Vancouver Index of Acculturation
GHSQ: The General Help-Seeking Questionnaire
